# How Do Red Blood Cells Die?

**DOI:** 10.3389/fphys.2021.655393

**Published:** 2021-03-15

**Authors:** Perumal Thiagarajan, Charles J. Parker, Josef T. Prchal

**Affiliations:** ^1^Center for Translational Research on Inflammatory Diseases (CTRID), Michael E. DeBakey Veterans Affairs Medical Center, Houston, TX, United States; ^2^Department of Pathology and Immunology, Baylor College of Medicine, Houston, TX, United States; ^3^University of Utah School of Medicine, Salt Lake City, UT, United States; ^4^Huntsman Cancer Institute, University of Utah, Salt Lake City, UT, United States

**Keywords:** red cell deformability, phosphatidylserine (PS) exposure, complement, Band 3, spleen

## Abstract

Normal human red blood cells have an average life span of about 120 days in the circulation after which they are engulfed by macrophages. This is an extremely efficient process as macrophages phagocytose about 5 million erythrocytes every second without any significant release of hemoglobin in the circulation. Despite large number of investigations, the precise molecular mechanism by which macrophages recognize senescent red blood cells for clearance remains elusive. Red cells undergo several physicochemical changes as they age in the circulation. Several of these changes have been proposed as a recognition tag for macrophages. Most prevalent hypotheses for red cell clearance mechanism(s) are expression of neoantigens on red cell surface, exposure phosphatidylserine and decreased deformability. While there is some correlation between these changes with aging their causal role for red cell clearance has not been established. Despite plethora of investigations, we still have incomplete understanding of the molecular details of red cell clearance. In this review, we have reviewed the recent data on clearance of senescent red cells. We anticipate recent progresses in *in vivo* red cell labeling and the explosion of modern proteomic techniques will, in near future, facilitate our understanding of red cell senescence and their destruction.

## Introduction

Under normal physiological condition, red cell concentration is maintained at a relatively constant value of ∼5 million per μL (4.52–5.90 in men and 4.10–5.10 in women) by equilibration of production and destruction. Red cells are produced when hematopoietic stem cells differentiate into erythroid precursors that subsequently mature into circulating erythrocytes. While the mechanisms involved in erythropoiesis are understood in detail, the processes that underlie physiological clearance of senescent red cells from the circulation are incompletely understood. Red cells have an average life span of about 120 days after which they are cleared by- phagocytosis by reticuloendothelial macrophages due to accumulated changes during their life span. Approximately 5 million erythrocytes (the average number per μl) are removed from the circulation every second.

During circulation, blood cells are exposed to constant physical and chemical stress. The static erythrocyte is a biconcave disk, 8–10 μm in diameter, 2.5 μm thick at the edge, and 1 μm thick at the center. This shape is well suited for gas transport, providing optimal surface area for oxygen and CO_2_ diffusion. However, red cells must pass innumerable times during their life span without rupturing through capillaries with diameters of approximately 2–4 μm. Physical stress encountered by red cells is especially severe when they traverse interendothelial slits of ∼ 2 μm in the venous sinus of the splenic red pulp. The remarkable pliability of the membrane allows to the red cell to undergo a complex transition from the biconcave shape to a parachute-like configuration through membrane folding and cytoplasmic reorganization during its flow in the microcirculation (Tomaiuolo and Guido, 2011). Following the completion of its journey through the microcirculation the red cells regain its efficient biconcave disk shape, however, repeated physical stress encountered in the microcirculation results in accumulated loss of deformability that adversely affect its life span (Carrell et al., 1977; Rifkind et al., 2019).

During oxygen delivery, a small fraction of the oxygen released from hemoglobin generates highly destructive reactive oxygen species (ROS) including superoxide. ROS oxidize hydroxylated sulfur groups (SH), leading to alterations in protein structure. One of the consequences of this process is transformation of the hemoglobin into to an insoluble product that precipitates as Heinz bodies. ROS also damages proteins in the red cells membrane, leading to greater membrane rigidity that further contributes to decreased red cells survival. In addition, ROS oxidizes ferrous iron (Fe^2+^) to ferric iron (Fe^3+^), converting hemoglobin to methemoglobin, which cannot bind oxygen. The red cells have robust redox mechanisms to quench the ROS and protect the hemoglobin and other components including membrane proteins, lipids, and cytoplasmic components from injury. To repair this damage and to allow oxygen to bind to hemoglobin, red cells metabolism must generate reduced nicotinamide adenine dinucleotide phosphate (NADPH) for antioxidant protection mediated principally by glucose-6-phosphate dehydrogenase (G6PD) activity. G6PD-deficient red cells have shorter *in vivo* survival (Sagiv et al., 2018). Red cells also generate nicotinamide adenine dinucleotide (NADH) that must be available to continuously reduce methemoglobin to hemoglobin. Glycolysis generates ATP to fuel red cell membrane integrity and transport pathways, or alternatively generation of ATP is bypassed (“energy shunt”) to form 2,3 DPG that enhances tissue oxygen delivery in the Rapoport Luebering pathway (Carrell et al., 1977; Rifkind et al., 2019). Several studies, based separation on density gradients, have shown as the cells age in the circulation the activity of several glycolytic enzymes decrease (Haram et al., 1991). The age-dependent decay of red cell enzymes may predispose to greater oxidant injury in senescent red cells. Several of these changes have been studied as tags for macrophage recognition and clearance of senescent red cells, including loss of membrane resulting in impaired deformability, oxidation of membrane proteins, appearance of neoantigens, exposure of membrane phosphatidylserine, changes in enzymatic activity, and decrease in the activity of the thrombospondin-1 receptor (CD47). Despite extensive study, the mechanisms that mediate senescent red cells clearance remain largely speculative (Piomelli and Seaman, 1993; Rifkind and Nagababu, 2013; Lew and Tiffert, 2017; Badior and Casey, 2018).

## Role of Spleen in Red Cell Clearance

The spleen is a lymphoid organ that functions primarily as a filter for the blood (Mebius and Kraal, 2005). The structure of the splenic microcirculation is optimized to remove defective red cells, blood-borne microorganisms, and cellular debris. Splenic blood flow consists of both open and closed sites of circulation. Splenic arterioles empty into a reticular meshwork called the red pulp that is rich in macrophages and not lined by endothelial cells. Consequently, erythrocytes transit the red pulp slowly under low shear stress to reach the splenic sinuses. The high density of macrophages in the red pulp results in contact with the red cells. Macrophages recognize damaged, deformed and senescent erythrocytes and remove them from circulation by phagocytosis. In addition, without damaging the red cell, macrophages remodel the cell by removing nuclear remnants (Howell-Jolly bodies) and other inclusions such as Heinz bodies (denatured hemoglobin caused by oxidative damage), Pappenheimer bodies (phagosomes containing excess iron), and siderocytes (iron granules that are not contained within hemoglobin). Endothelial cells of the splenic sinuses are separated by interendothelial fenestrations ∼2 μM in diameter that are lined by basement membrane. The pliability of the membrane allows the red cells to traverse these tight spaces to reach the splenic sinuses. Erythrophagocytosis of senescent red cells readily occurs *in vivo* but incubation of senescent cells *in vitro* with cultured splenic macrophages does not induce erythrophagocytosis suggesting that the characteristics of splenic architecture are necessary for clearance of aged red cells (Gottlieb et al., 2012). How precisely the spleen senses senescent red cells is not clear. Altered deformability may not be the principal mechanism. Southeast Asian ovalocytosis or Melanesian ovalocytosis is an inherited disorder characterized by abnormal red cell membrane rigidity due to deletion of nine amino acids within the cytoplasmic domain of the most abundant red cell cytoskeleton protein, Band 3 [anion exchanger 1 (AE1), encoded by *SLC4A1*)] (Hadley et al., 1983; Saul et al., 1984). Despite decreased membrane deformability, these ovalocytes circulate normally without abnormal sequestration by the spleen (Safeukui et al., 2018). Klei et al. (2020) reported that *in vivo*, in the splenic microcirculation, senescent red cells adhere to splenic macrophages via laminin-α5. Consequently, some of the red cells undergo hemolysis. In this setting, red pulp macrophages preferentially engulf erythrocyte ghosts over intact red cells. The authors hypothesized that phagocytized red cell ghost are more rapidly degraded than their intact counterparts, and that scavenging heme from within the splenic circulation is more efficient for iron recycling than extraction from phagolysosomes. However, this hypothesis is not supported by the observation that splenectomy does not affect red cell lifespan or iron metabolism (Anosa, 1976). As the red cells age in the circulation, they lose hemoglobin and membrane. This loss occurs due to shedding of hemoglobin-containing microvesicles – a process facilitated by the pitting action spleen (Willekens et al., 2003). While the spleen is essential for remodeling of erythrocytes and clearing of defective red cells, more needs to be learned about splenic contribution to physiological clearance of senescent erythrocytes. It is very likely there are other sites for senescent red blood cell clearance.

## Methods to Isolate Senescent Red Cells

A major limitation in the study of the processing of senescent cells is the absence of a reliable technique to isolate the target cells just before clearance. A variety of techniques have used including separation based on density (Borun et al., 1957; Cohen et al., 1976), differential agglutination (Allison and Burn, 1955), osmotic fragility (Marks and Johnson, 1958), induction of bone marrow arrest (Reissmann and Ito, 1966; Van Dilla and Spalding, 1967), and isolation of red cells from patients with acute aplastic anemia (Linderkamp et al., 1993). The most commonly used method of separation is based on density. When a cohort of red cells, labeled with radioactive iron (^53^Fe), were centrifuged, radioactivity was detected primarily in the low-density red cells concentrated near the top of a centrifuged cell sample. Over time, radioactivity progressively shifted toward higher density fractions at the bottom of centrifuged sample. The conclusion from these studies was that red cells become progressively denser as they age within the circulation and that density-dependent cell separation methods can be used to isolate cell populations based on age related density. Other studies showed putatively older cells separated based on density had decreased sodium efflux (Bernstein, 1959; Seaman et al., 1980; Hentschel et al., 1986), resulting in progressive dehydration during the red cell life span. However, concerns have been raised about using density as the sole criterion for red cell aging. Studies have shown that labeled cohorts of human red cells do not increase in density uniformly throughout their life-span. Rather, during the latter half of the life-span of the cohort, density becomes diffusely distributed (Bernstein, 1959) with only a slight increase in dense cells. The Ganzoni hypertransfusion protocol suppresses endogenous erythropoiesis by maintaining polycythemia by transfusion in mice or rats such that progressively older cells can be obtained for analysis (Ganzoni et al., 1971). Results from using this model showed that older red cells are not denser despite their shorter life span following autologous transfusion (Mueller et al., 1987). Other concerns about density fractionation such as low-resolution of gradient separations and the confounding effects of reticulocytes in the less dense fractions have been raised (Clark, 1988). Furthermore, some senescent cells can undergo terminal density reversal shortly before clearance by poorly understood mechanisms further complicating the correlation between cell density and senescence (Lew and Tiffert, 2013). Nevertheless, a studies continue to be, and have been performed, based on red cells density gradient separation.

Clinical studies have also given conflicting information. A human study showed that the denser fractions of ^56^Cr labeled cells disappeared more quickly than red cell in lighter fractions (ten Brinke and de Regt, 1970). However, Linderkamp et al. (1993) examined the red cells of children with transient erythroblastopenia of childhood, a disorder causing temporary cessation of erythropoiesis. The red cells from these patients exhibited no changes in density and only a small fraction of deformable red cells was diminished. The tag for macrophage removal of senescent red cells appears to behave in a binary fashion, becoming expressed at the time when the cell reaches the end of its life span.

An alternative method to identify senescent red cells based on biotinylation of cell surface proteins have been developed by Dale et al. Dale and Norenberg (1990) and Waugh et al. (1992). Red cells are labeled *ex vivo* and infused into animals or labeled by intravenous injection of biotin *in vivo*. At various time intervals, the biotinylated red cells can be isolated using an avidin matrix. Studies of biotinylated rabbit red cells showed that old cells had both decreased surface area (10.5%) and volume (8.4%) resulting in little change in surface-to-volume ratio during aging. The aged cells were found to have normal membrane elasticity with only a minority of the cells being recovered from denser fractions following centrifugation. A rapid and robust method compliant with good practice guidelines was developed to generate biotin-labeled red cells for clinical research (de Back et al., 2018). Furthermore, a stable isotope-based mass spectrometric method using non-radioactive 15N-glycine for cohort labeling has been developed and it do not involve membrane modification or radioactivity (Browne et al., 1993). This method show good correlation with the biotin method (Khera et al., 2015). We expect that application of these procedures will advance our understanding of the molecular basis of red cell aging in humans by providing a uniform approach to analysis of erythrocyte physiology and pathophysiology.

## Clearance Mechanisms Based on Changes in Membrane Proteins

Band 3 (*SLC4A1*) is the most abundant integral membrane protein in the red cell with more than a million copies per cell. It is a 911-amino acid glycoprotein and the founding member of the anion exchanger gene family (AE-1). In the membrane, Band 3 associates with a number of other membrane proteins including the Rh complex, glycophorins, and CD47. On the cytoplasmic side, it attaches to the membrane cytoskeleton through interactions with Band 4.2 and ankyrin, and this complex is a component of the cytoskeleton that is required to maintain the shape and integrity of red cells. In addition, its carboxyl terminus is attached to carbonic anhydrase, which catalyzes the conversion of CO_2_ to HCO_3_− and H+(Vince and Reithmeier, 1998). Band 3 mediates the electroneutral exchange of bicarbonate for plasma chloride allowing CO_2_ to be transported as HCO_3_− in the plasma. This process is reversed in the lungs. The crystal structure of transmembrane domain of Band 3 has been solved, providing insights into its functional interactions (Hatae et al., 2018). Deoxyhemoglobin binds to Band 3 and this interaction is implicated in sensing changes in oxygen tension and controlling red cell deformability thereby capillary velocity during hypoxia (Zhou et al., 2019). Furthermore, this interaction is also implicated in senescence induced clearance. Alteration in Band 3 has been studied as senescent tag for macrophage recognition and clearance if senescent red cells. Oxidant stress-mediated denaturation of hemoglobin produces hemichromes that accumulate in the cytoplasm and copolymerize with the cytoplasmic domain of Band 3, forming an insoluble macromolecular aggregate (Low et al., 1985; Kannan et al., 1988). Oxidation of hemichrome-mediated clustering of Band 3 is thought to expose neoantigens that are recognized by naturally occurring antibodies and cleared by macrophages. In addition to hemichrome-induced clustering, calcium-dependent proteolytic degradation of Band 3 has been postulated to expose a senescent tag (Kay et al., 1986; Schwarz-Ben Meir et al., 1991). Furthermore, Band 3 clustering has been proposed to bind to an endothelial cell receptor for products of advanced glycation (Wautier and Wautier, 2020).

Naturally occurring antibodies (39) have germline immunoglobulin sequences that are polyreactive, binding with low affinity to multiple epitopes (Turman et al., 1991). Numerous functions have been attributed to these antibodies, including serving as the first line of defense against pathogens, clearance of cellular debris, and recognition of oxidation-specific epitopes in lipids and proteins. Naturally occurring antibodies that bind to clustered Band 3 or neoantigens in Band 3 have been proposed as mediators of senescent red cell clearance (Kay, 1985; Lutz, 2012). Studies by Kay et al. suggested that senescent antigen generation is the result proteolytic cleavage of Band 3 (Kay and Goodman, 1984). Old red cells were reported to have autologous serum antibodies bound to discrete regions of Band 3 localized to amino acids 538–554 and 778–827 (Kay and Lin, 1990). In addition to antibodies against Band 3, naturally occurring antigalactosyl antibodies have been postulated to bind these cryptic antigens exposed at senescence (Galili et al., 1986). However, there is no alteration in red cell clearance in agammaglobulinemic mice, questioning the physiological significance of antibody-mediated clearance of senescent red cell (Connor et al., 1994; Bratosin et al., 2002; Hudson et al., 2017). Advances in proteomics should further refine analysis of changes in Band 3 during aging *in vivo* (Bosman et al., 2008).

Band 4.1 is required for maintaining red cell shape by regulating the mechanical properties of deformability through lateral interactions with spectrin, actin and glycophorins. On SDS-PAGE electrophoresis, it appears as two distinct bands with molecular weights of 80 kDa (4.1a) and 78 kDa (4.1b). Inaba et al. (1992) have shown that the basis of the difference in molecular weight is post-translational deamidation of two carboxy-terminal asparagine residues (Asn478 and Asn502), which decreases the electrophoretic mobility of the protein. Deamidation may induce conformational changes that alter the functional characteristics of Band 4.1. The 78 kDa form (4.1b) is the major component in younger cells and reticulocytes (Sauberman et al., 1979). Mueller et al. (1987) analyzed the membrane proteins in young and old red cells by maintaining mice in a state of continuous erythropoietic suppression for up to 8 weeks using a serial hypertransfusion protocol. They showed that during *in vivo* aging, there is an increase in the ratio of 4.1a:4.1b and proposed this change in ratio as a measure of *in vivo* senescence (Mueller et al., 1987). The ratio of 4.1a:4.1b correlates with the average life span of red cells in other species, but whether the change in ratio contributes mechanistically to clearance of senescent red cells remains speculative.

CD47 is a heavily glycosylated cell surface protein belonging to the immunoglobulin superfamily that is present in red cells in complex with Rh proteins (Oldenborg, 2004). CD47 binds to its cognate receptor, signal regulatory protein alpha (SIRPα), on macrophages and suppresses phagocytosis by inhibiting inside-out activation of integrin signaling (Morrissey et al., 2020). CD47 deficient red cells are rapidly cleared from the peripheral circulation by splenic macrophages in a process that is independent of complement and antibodies (Oldenborg et al., 2000). Physiologically, CD47 functions to prevent autologous cells from undergoing phagocytosis (Jaiswal et al., 2009). Changes in CD47 have been investigated in relation to erythrocyte storage and senescence. CD47 was reported to be lost from human erythrocytes during storage (Anniss and Sparrow, 2002), and the density of CD47 was reported to decrease during aging of murine erythrocytes surface (Khandelwal et al., 2007), suggesting that reduced expression of CD47 facilitates phagocytosis of stored and senescent red cells. However, Rh(null) cells with reduced CD47 expression do not show heightened interaction with monocytes (Arndt and Garratty, 2004). Furthermore, in autoimmune hemolytic anemia, CD47 expression is normal (Ahrens et al., 2006).

## Clearance Mechanisms Based on Changes in Membrane Carbohydrates

Desialylation of membrane proteins was among the first mechanisms postulated to account for clearance of senescent red cell (Aminoff et al., 1977). Most erythrocyte membrane proteins are rich in sialic acid, which gives the cell a negative charge. It was hypothesized that progressive loss of sialic acid occurred as red cells age thereby providing a marker of senescence and a mechanism for recognition and clearance aged erythrocytes. Despite demonstration that enzymatic desialylation of red cells results in rapid clearance, sialic acid content does not change significantly as erythrocytes age (Shinozuka et al., 1988).

Klei et al. (2018) postulated a novel mechanism involving sialic acid in the clearance of senescent red cells. Lutheran/basal cell adhesion molecule (Lu/BCAM) is a transmembrane adhesion molecule that interacts with the extracellular matrix protein, laminin. This interaction is inhibited by sialic acid moieties of glycophorin C, a minor erythrocyte membrane glycoprotein (Jaskiewicz et al., 2018). Age related loss of glycophorin C sialic acid was hypothesized to contribute to clearance of senescent red cells by decreasing the inhibition of Lu/BCAM with laminin.

## Clearance Mechanisms Based on Phosphatidylserine Exposure

Asymmetrical distribution of phospholipids is a common property of all mammalian cells. In the case of red cells, anionic phospholipids reside in the inner leaflet while neutral or zwitterionic phospholipids predominantly comprise the outer leaflet (Bretscher, 1972; Verkleij et al., 1973; Connor et al., 1992). Phosphatidylserine exposure in the outer leaflet is the basis of recognition of apoptotic cells by the macrophages (Penberthy and Ravichandran, 2016). Schroit et al. (1985) reported that red cells enriched with phosphatidylserine analogs (by insertion of controlled amounts of fluorescent phosphatidylserine analogs into mouse red cells) on the outer surface are cleared five times faster than controls. These cells accumulated in splenic macrophages and hepatic Kupffer cells. Cell clearance depended on the amount of exogenously inserted phosphatidylserine, and more rapid clearance was when the cells contained as little as 1% phosphatidylserine (Schroit et al., 1985). However, clearance was incomplete, and this observation was attributed to aminophospholipid translocase activity, which continuously pumps phosphatidylserine to the inner leaflet of the circulating cells, thereby preventing red blood cell recognition by macrophages. Using annexin V binding to quantify phosphatidylserine exposure by biotin labeled red cells, Boas et al. (1998) reported greater binding for aged red cells, with the extent of phosphatidylserine expression paralleling the rate at which the biotinylated red cells were removed from circulation. Other studies, however, have not shown a greater phosphatidylserine in aged red cells compared to their younger counterparts (Wesseling et al., 2016). One explanation for these disparate observation is that phosphatidylserine-expressing red cells are removed from the circulation by macrophages at a rate that makes them undetectable (Schroit et al., 1985; Dasgupta et al., 2008).

Conceivably disturbances in mechanisms involved in the maintenance of membrane phospholipid could contribute to red cell senescence. P-type ATPases (flipases) catalyze the transport of phospholipids from the outer to the inner leaflet, Sebastian et al. (2012) while ABC ATPases (flopases) catalyze the transport of lipids from the inner to the outer leaflet (Coleman et al., 2013). Another enzyme, scramblase, that facilitates movement of lipids between both leaflets, has been identified. In contrast to ATP-dependent lipid translocases, scramblases are generally non-selective and energy independent, being activated by an increase in intracellular Ca^2+^ (Pomorski and Menon, 2016). Lipid scrambling changes the architecture of the bilayer, promoting exposure of phosphatidylserine and release of extracellular vesicles (Nagata et al., 2016; Whitlock and Hartzell, 2017).

A number of mechanisms have been proposed to explain phosphatidylserine exposure during red cells senescence. Oxidation of phosphatidylserine alters its ability to act as a substrate for aminophospholipid translocase, which transports phosphatidylserine from the outer to inner leaflet (Tyurina et al., 2000). Aging of erythrocytes increases both membrane lipid peroxidation (Ando et al., 1995) and oxidation of phosphatidylserine, consequently allowing phosphatidylserine to persist in the outer layer for macrophage recognition. The cytoskeleton in an important component of the process by which lipid asymmetry is maintained. Phosphatidylserine interacts with the major membrane cytoskeletal protein, spectrin (Kunzelmann-Marche et al., 2001). Disruption of membrane-cytoskeletal interactions, due to senescence-induced aggregation of Band 3, may result in phosphatidylserine exposure. In a mouse model, widespread thrombosis and severe hemolysis due to increased phosphatidylserine exposure was observed in Band 3 null red cells (Hassoun et al., 1998). However, in hereditary spherocytosis, erythrocytes fully conserve lipid asymmetry despite abnormalities in membrane skeleton components (Calvez et al., 1988; Kuypers et al., 1993). This observation suggests that the contribution of cytoskeletal proteins to regulation of phosphatidylserine is insignificant.

An increase in intracellular Ca^2+^ in red cells activates scramblase, exposing phosphatidylserine similar to that observed in nucleated cells during apoptosis (Fadok et al., 2001). Storage of human red cells under blood bank conditions resulted in a small increase in Ca^2+^ permeability (Larsson et al., 2016), but an age related mechanism that increases red cell calcium has not been identified (Khandelwal and Saxena, 2008). On the other hand, clustering of Band 3 has bee reported to induce externalization of phosphatidylserine by a calcium- and oxidation-independent mechanism (Koshkaryev et al., 2020).

Eryptosis is the process of cell shrinkage and exposure of phosphatidylserine due influx of calcium ions that activates a scramblase resulting in redistribution of phospholipids in both leaflets (Lang et al., 2008). Eryptosis may contribute to red cell clearance in diseased states, but its contribution to senescence-associated clearance is speculative.

Physiological processes that delay senescence may contribute to hypoxia-induced erythrocytosis Tang et al. (2019) reported a reduction in phosphatidylserine, cytosolic Ca^2+^, and an increase in CD47 in red cells exposed to chronic hypoxia, and red cells produced *in vivo* under hypoxic conditions had a longer lifespan than cells produced under normoxic conditions. The studies provided the first evidence suggesting that longer red cell survival, in addition to hypoxia stimulation of erythropoiesis contributes to hypoxia-induced erythrocytosis.

Lactadherin, also called milk fat globule epidermal growth factor 8, is an opsonin that binds to phosphatidylserine expressing cell, including red cells (Hanayama et al., 2002; Dasgupta et al., 2008). It has a phosphatidylserine binding domain, as well as an Arginine-Glycine-Aspartic acid (RGD) motif in one of its epidermal growth factor domains which mediates binding to integrins (Andersen et al., 1997; Hanayama et al., 2002). Lactadherin-mediated erythrophagocytosis of phosphatidylserine expressing cells by integrins is proposed as a mechanism for clearance of senescent red cells by activated endothelial cells (Fens et al., 2012). However, red cell survival is normal in lactadherin deficient mice, and as discussed above, whether senescent red cells express greater amounts of phosphatidylserine is an issue of active debate (Dasgupta et al., 2008; Wesseling et al., 2016).

Although speculative, other phosphatidylserine binding proteins expressed by macrophages including CD36, SR B1, CD68, CD14, Mer, LOX-1 Bai1, TIM-4 RAGE, and stabilin-2 may be involved in clearance of senescent red cells, Bratton and Henson (2008), Park et al. (2008), and Ravichandran (2010).

### Neocytolysis

Physiologic response to hypoxia is the stimulation of red blood cell production. Hypoxia-inducible factors (HIFs) orchestrate response to hypoxia and HIF-2 is the principal regulator of erythropoietin (EPO) production in kidney as underscored by genetic studies in human populations that live at high-altitude and by mutational analysis of patients with familial erythrocytosis (Prchal, 2015). Upon the rapid return to normoxia, the secondary erythrocytosis is overcorrected, as the accumulated, newly formed red cells undergo preferential destruction. This process, termed *neocytolysis*, was originally observed in astronauts returning to earth after living in a zero gravity environment (Rice et al., 2001). Return to normoxia from hypoxia results in generation of reactive oxygen species from increased mitochondrial mass that correlates with decreased expression of *Bnip3L* transcripts, a hypoxia regulated gene (Sandoval et al., 2008; Song et al., 2015). Bnip3L mediates removal of reticulocyte mitochondria that generate increased reactive oxygen species accompanied by reduced catalase activity mediated by hypoxia-regulated miR21 (Song et al., 2015). Rapid changes in hematocrit in human newborns also suggest that neocytolysis also occurs after birth. The hypoxic fetus has erythrocytosis at birth, but the neonate rapidly overcorrects its elevated red cell mass and becomes anemic in first 2 weeks of life (Christensen et al., 2013).

## Pathological Red Cells Clearance

In contrast to our limited understanding of the physiological red blood cell clearance of senescent red cells, the mechanisms involved in removal of abnormal erythrocytes (hemolysis) are understood in greater details. Premature destruction can occur in the circulation by lysis with the release of hemoglobin into the plasma (intravascular hemolysis) or by the macrophages in the spleen and liver (extravascular hemolysis) with little release of hemoglobin. The spleen plays a major role here. Increased splenic clearance occurs due to injuries extrinsic events (immunological targeting, mechanical or chemical injuries) or due to intrinsic defects in red cells (due to inherited defects in red cells cytoskeleton or enzymes).

## Red Cell Deformability and Splenic Clearance

Red cells with reduced deformability are unable to negotiate through narrow endothelial slits in the human spleen. Consequently, they are retained in the splenic cords and eventually destroyed by red pulp macrophages. The principal determinants of the red blood cell deformability are the ratio of cell surface area to volume (determined by the shape), intracellular viscosity (determined the physical properties of hemoglobin), and membrane elasticity (determined by rheological properties of the membrane). As discussed earlier, red cells traverse the interendothelial slit in splenic sinusoids. When normal deformity is compromised, sustained elongation results in loss of membrane due to vesiculation (Li et al., 2018). Premature destruction occurs in many membrane disorders including hereditary spherocytosis, ovalocytosis, and pyropoikilocytosis. In addition to intrinsic membrane defects, the red cell membrane can be damaged by abnormalities in microcirculation due intravascular fibrin deposition and abnormal shearing due artificial heart valves, or severe aortic stenosis. The fragment erythrocytes are rapidly removed by the reticuloendothelial system. Collectively these processes are called microangiopathic hemolytic anemia.

## Oxidant Injury and Red Cell Clearance

Red cells efficiently transport oxygen throughout their lifespan unless they are damaged by ROS. Consequently, they have effective mechanism to quench ROS. The red cell spends a significant amount of energy to keep NADP in its reduced form (NADPH), thereby maintaining a readily available pool of electron donors to reduce ROS. Depletion of NADPH can occur in the G6PD deficiency, or structural hemoglobin abnormalities that predispose to hemoglobin oxidation, or exposure to oxidant drugs. Oxidation of hemoglobin alters the quaternary structure allowing them to precipitate within the red cell and to form aggregates called Heinz bodies. Heinz bodies attach to the red cell membrane decreasing deformability, thereby rendering the affected cells susceptible to engulfment by sinusoidal macrophages of the spleen and liver to membranes decreases the deformability and other physical properties pliable of red cell membrane, rendering them to engulfment by macrophages in rich sinusoids of spleen and liver. Macrophage-mediated removal of Heinz bodies leaves a defect in the erythrocyte membrane structure that may be seen as bite cells on microscopic examination of the peripheral blood smear.

## Antibody–Mediated Red Cells Destruction

Antibody-mediated intravascular hemolysis occurs due to complement activation by the classical pathway. IgM antibodies fix complement more avidly than IgG antibodies because of their they are pentavalent rather than bivalent. However, two IgG molecules in close proximity on the RBC surface can also activate complement. Hence, the antigen density is a critical determinate of complement activation in IgG-mediated immune hemolytic anemia (Garratty, 2008). As the macrophages do not express IgM Fc receptors (Kubagawa et al., 2009), IgM-mediated red cell destruction is mediated directly through complement induced damage and indirectly through macrophage clearance of complement opsonized cells. Immune complexes activate the classical complement pathway by binding the C1q portion of the C1 complex. Exposure of the collagen-like regions of C1q makes it recognizable by macrophage complement receptor 1 (CR1) (Eggleton et al., 2000), however, C1q opsonization does not contribute significantly to the pathophysiology of immune-mediated hemolytic anemia. Rather, during complement activation, the third component of complement (C3) is cleaved to C3b, which can bind covalently to cell surface carbohydrate and peptide moieties. Bound C3b is rapidly cleaved to an inactivated form, iC3b. Macrophages have receptors for C3b (CR1) and iC3b (CR3). Unlike FcγRs, which are constitutively active, the phagocytic function of CR3 requires activation through a distinct pathway (Allen and Aderem, 1996; Caron and Hall, 1998). Cell bound iC3b is rapidly degraded enzymatically to C3dg and C3d. The receptor for C3dg and C3d is CR2, expressed primarily by B lymphocytes. C3d/C3dg binds to CR2 with low affinity. Therefore clearance of C3d/C3dg opsonized red cells is inefficient, such that their lifespan is shortened less than C3b and iC3b coated cells, and red cells opsonized by C3d/C3dg can be found circulating in the peripheral blood of patients with immune hemolytic anemia, particularly in patients with cold agglutinin disease (Aderem and Underhill, 1999).

IgG-mediated extravascular hemolysis is mediated by macrophages in the spleen and liver (Eggleton et al., 2000). IgG antibodies bind red cell antigens, and the Fc portion of the bound immunoglobulin is recognized by specific macrophage receptors that mediate phagocytosis of the opsonized erythrocytes. There are three types of Fcγ receptors that activate phagocytosis, FcγRI (CD64), FcγRIIA (CD32a), and FcγRIII (CD16). Phagocytosed red cells are targeted to phagolysosomes (Mosser and Zhang, 2011). FcγRI has the highest affinity for IgG molecules, while FcγRIIA and FcγRIII have lower affinities (Bruhns et al., 2009). Furthermore, there are four subtypes of IgG – IgG1, IgG2, IgG3, and IgG4 with varying affinities for FcγRs (Vidarsson et al., 2014). FcγRI is a dimer consisting of a ligand-binding α-chain and a signal-transducing γ-chain, which carries immunoreceptor tyrosine based activating motifs (ITAMs) (Bournazos et al., 2016). ITAM-induced phosphorylation activates signaling pathways, including phosphatidylinositol kinase and MAP kinase that induces efficient erythrophagocytosis such that little free hemoglobin is released into the circulation. In addition to activating receptors, macrophages also express FcγRIIB, which is an inhibitory receptor that transmits signals through an immunoreceptor tyrosine-based inhibitory motif (ITIM) contained in cytoplasmic domain of the receptor. Intravenous immunoglobulin (IVIG) is a modestly effective treatment for autoimmune hemolytic anemia (Flores et al., 1993). Although other mechanisms contribute, the therapeutic activity of IVIgG appears to be mediated in part through binding to FcγRIIB.

## Conclusion

This review provides an overview of our current knowledge of the mechanisms involved red cell clearance. Despite the plethora of investigations, our understanding of the molecular details of red cell clearance is incomplete. Recent progresses in *in vivo* red cell labeling and availability of novel proteomic techniques should provide the means to enhance our understanding of the processes that underlie red cell senescence.

## Author Contributions

All authors listed have made a substantial, direct and intellectual contribution to the work, and approved it for publication.

## Conflict of Interest

The authors declare that the research was conducted in the absence of any commercial or financial relationships that could be construed as a potential conflict of interest.
